# Circumcision With the Plastibell Technique: A Descriptive Case Series

**DOI:** 10.7759/cureus.30601

**Published:** 2022-10-23

**Authors:** Nisar Ahmed, Zaka Ullah Jan, Muhmmad D Yasin, Mahmud Aurangzeb

**Affiliations:** 1 General Surgery, Khyber Medical College, Peshawar, PAK

**Keywords:** dorsal penile nerve block, local anaesthesia, male circumcision, circumcision, plastibell

## Abstract

Background

The Plastibell technique is one of the most commonly performed procedures used for male circumcision (MC) and is in practice throughout the world. It is a procedure done under local anaesthesia, mostly on infants. The results of the technique have been evaluated in many studies throughout the world. We have done a series of cases and present the results of our retrospective descriptive study. The objective of this study was to describe the outcomes of circumcision performed via the Plastibell technique.

Materials and methods

This retrospective descriptive study was conducted at the Surgical A Unit, Khyber Teaching Hospital, Peshawar from July 2013 to June 2021. Clinical records of the infants who underwent circumcision were collected. Infants under the age of six months brought by their parents were included in the study. Infants whose parents requested methods other than Plastibell and infants with bleeding disorders or a family history of such disorders were excluded from the study. The indication for circumcision was for religious reasons in all cases. Post-operative complications were noted in all cases.

Results

A total of 364 male babies under the age of six months (mean age 43.5±15 days) underwent circumcision with the Plastibell technique. The mean operative time was 11.3±3.7 minutes. The time it took for the ring to fall off was 7.8±3.04 days. In one case, primary haemorrhage required exploration and diathermy of the bleeder. Oedema occurred in 76(20.8%) of the babies. Adhesions of the foreskin with the glans were formed in 3(0.82%) cases.

Conclusion

Male circumcision is one of the oldest surgical procedures performed. Several methods are in practise in this regard. The results of our study showed that circumcision with the Plastibell method is safe and has fewer side effects.

## Introduction

Male circumcision (MC) is one of the oldest surgical procedures performed and has traditionally been practised throughout most of Africa, Asia, Australia, large parts of South and Central America, and smaller areas of North America [1]. Various techniques of MC have been described, the commonest being the sleeve technique, the dorsal slit technique, and the use of certain instrumental techniques. Among the latter techniques, the Plastibell, Gomco clamp, and Mogen clamp techniques have been widely used [2].

The Plastibell technique has gained widespread popularity because of its ease of use, fewer complications, and requirement of local anaesthesia instead of general anaesthesia [3]. In this technique, a tight ligature is tied around the foreskin, drawn over a grooved plastic ring commonly known as the ’Plastibell’, and the skin distal to the ligature is excised. The ligature results in a circumferential line of ischemic necrosis and the Plastibell falls off within the course of a few days.

The Plastibell technique is a safe procedure with reproducible results [2,4-6]. The results are better in neonates and infants as compared to children [7]. In this study, we evaluated the data of the boys who underwent circumcision with the Plastibell technique at our unit.

## Materials and methods

This descriptive study was conducted at the Surgical A Unit, Khyber Teaching Hospital, Peshawar from July 1, 2013 to June 30, 2021. Clinical records of the infants (male babies aged less than one year) who underwent circumcision were collected. The Institution Research and Ethical Review Board (IREB) of Khyber Medical College, Peshawar, approved this study with approval number 390/DME/KMC. Infants under the age of six months brought by their parents were included in the study. The infants whose parents requested methods other than Plastibell and infants with bleeding disorders or a family history of such disorders were excluded from the study. Detailed informed and written consent was obtained from the parents.

**Figure 1:**
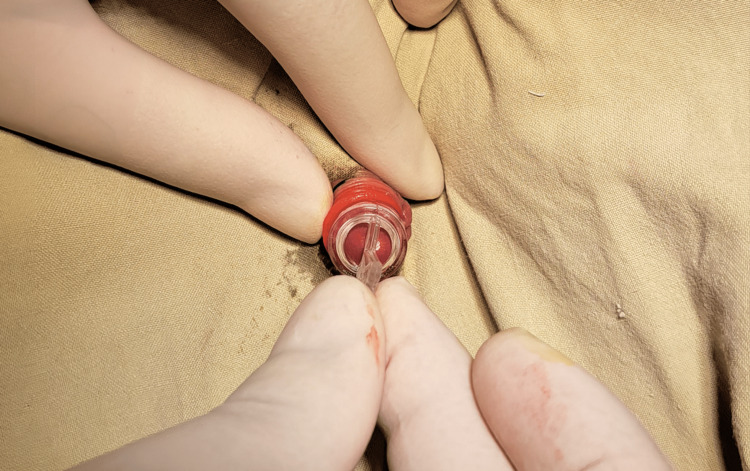
Prepuce retracted and appropriate size Plastibell selected

The procedure was performed under local anaesthesia. A penile ring block was performed using 1 ml of 2% lignocaine without adrenaline, and the baby was given back to the mother to wait for 15 minutes. The patients (infants or neonates) were placed in the supine position. After prepping the skin with povidone-iodine solution and draping with sterile drapes, the foreskin was retracted back and adhesions between the foreskin and glans were gently separated until the subcoronal sulcus. The residual smegma was carefully cleaned. Plastibell of an appropriate size was selected at this point (Figure 1). 

**Figure 2:**
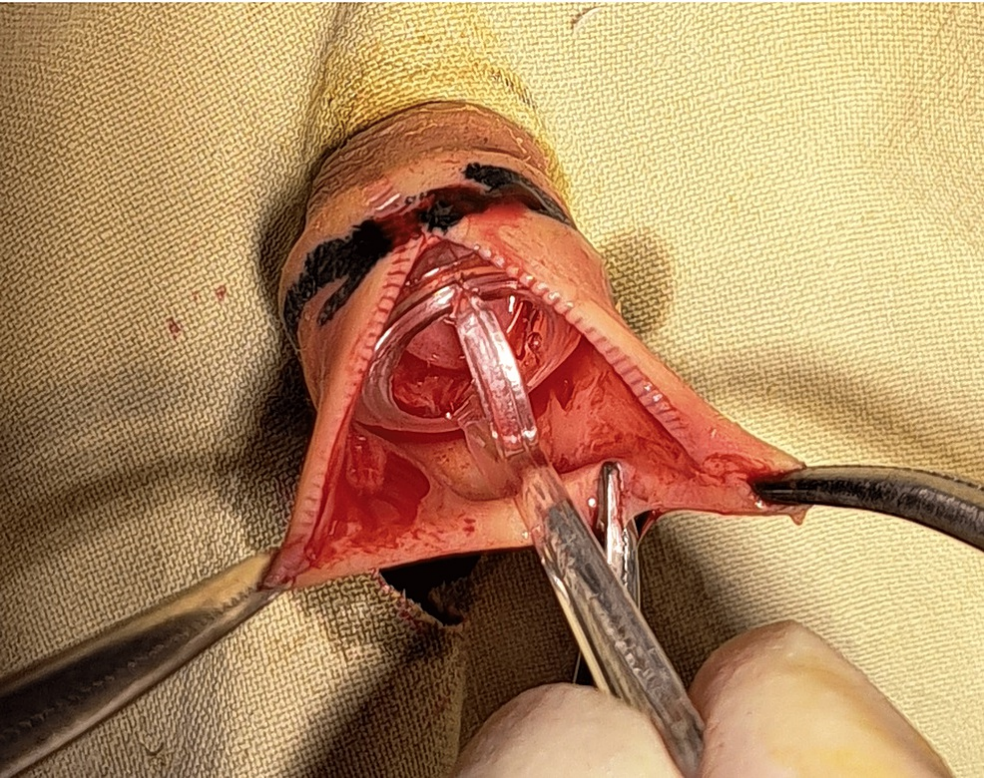
Dorsal slit done and Plastibell applied

**Figure 3:**
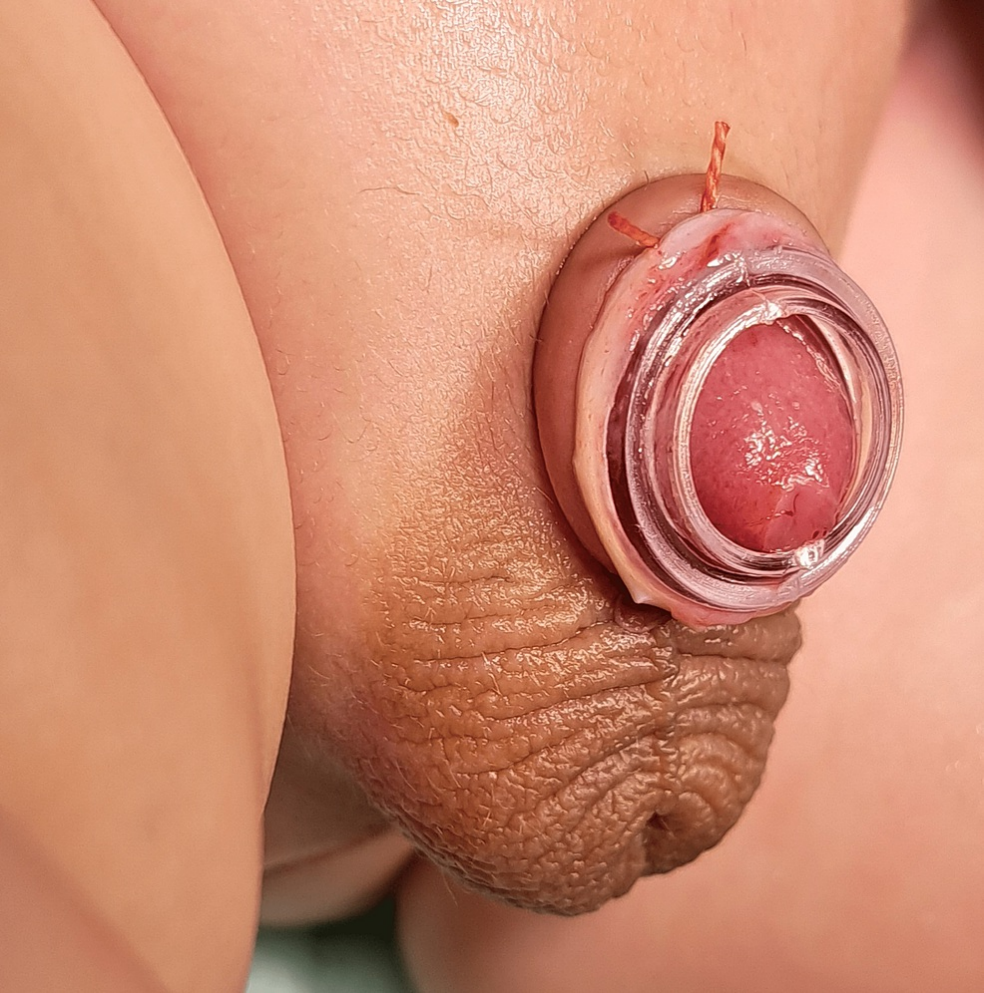
At the end of the procedure with Plastibell in place

The foreskin was drawn to the normal position and haemostats were applied to the tip of the foreskin at 3 and 6 o’clock positions. A dorsal slit was performed at the 12 o’clock position and the Plastibell was inserted. A ligature was carefully placed and tightened snugly at the previously marked skin site. The skin distal to the ligature was carefully incised and removed. The handle of the Plastibell was carefully disconnected from the ring (Figures 2-3).

**Figure 4:**
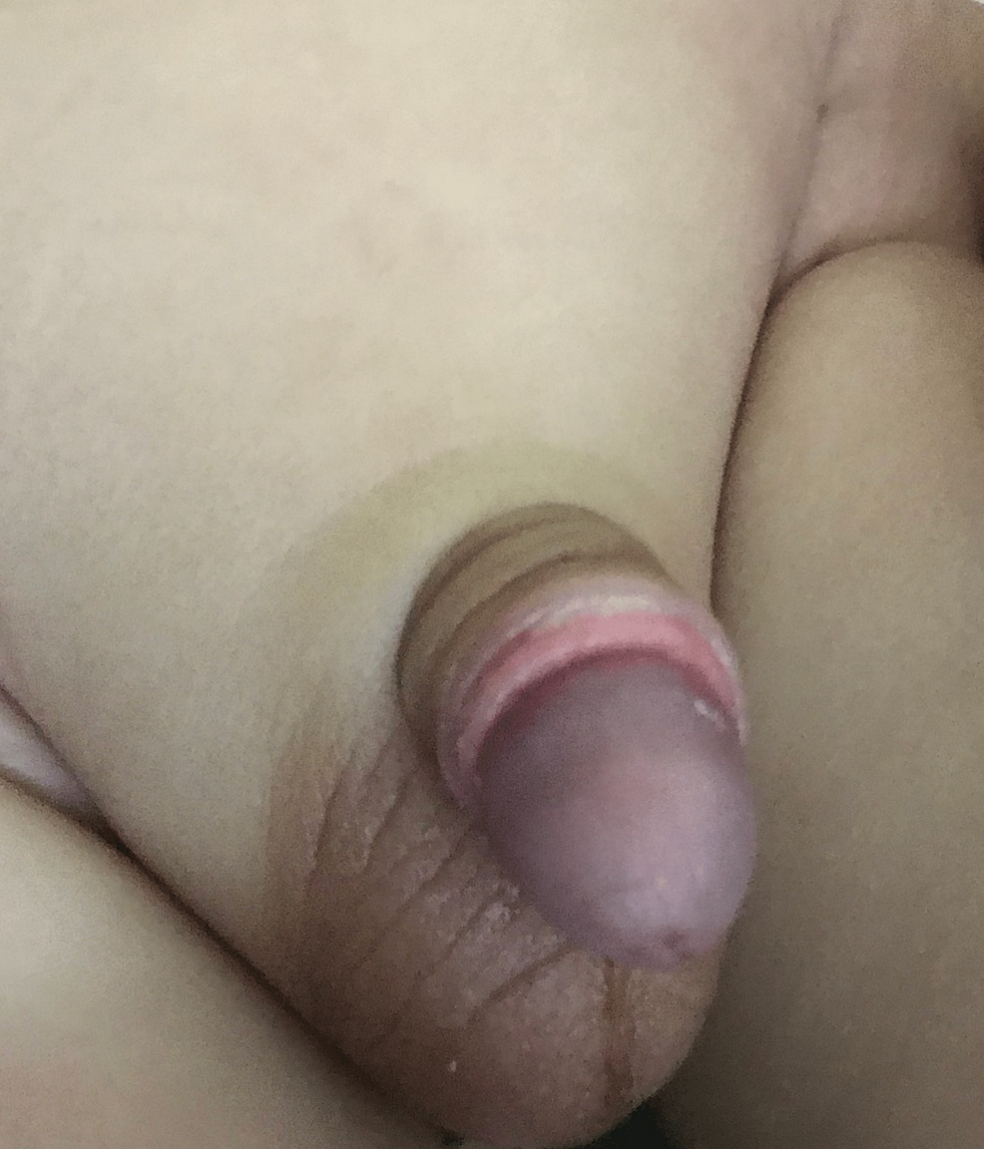
Follow up at two weeks after application of Plastibell

The parents were instructed about the postoperative care of the baby and were advised to bring him for follow-up after 10 to 14 days (Figure 4). Analgesia with paracetamol drops (20 mg/kg) q6h for three days was given to all patients. The use of antibiotics was discouraged in all patients.

## Results

A total of 364 records were identified that had at least one follow-up visit. The mean age of the babies was 43.5$\pm$15 days. The indication for circumcision was for religious reasons in all the cases. The mean operative time was 11.3$\pm$3.7 minutes. The mean time from the surgery to the ring detachment was 7.83$\pm$04 days.

Among the early complications, primary haemorrhage requiring re-exploration and diathermy of the bleeding point was noted in one case. Parents of 76 (20.8%) babies were worried about the presence of oedema at the circumcision site and they were reassured. Adhesions between the suture line and the glans penis (synechae) were formed in 3 (0.82 %) cases (Table 1).

**Table 1:** Results and early post-operative complications

Mean age	43.5± 15 days
Mean operative time	11.3±3.7 minutes
Mean time from operation to fall of ring	7.8±3.04 days
Primary haemorrhage	1(0.27%)
Oedema at the circumcision site	76(20.8%)
Synechae formation	3(0.82%)

## Discussion

Male circumcision is done with a variety of techniques throughout the world. The indications for MC are mostly religious and social needs. The potential benefits of circumcision include a reduction in the risk of UTI, cancer, phimosis, and cervical cancer for the female partner [8].

The Plastibell technique has the advantage of being a simple, haemostatic technique without the need for general anaesthetic [3]. The technique has been adopted worldwide. The success has been validated in numerous studies [2,4,6,8]. In most of the studies, the choice of anaesthetic/analgesic has been penile nerve block [9-13], although no gold standard procedure to address the pain in circumcision has been established [14]. In our study, the choice of anaesthetic was 2% Lignocaine with 1 ml solution infiltrated as a penile ring block. There was difficulty in assessing the adequacy of analgesia in most patients, though the cry at the incision time and suture tightening time were considered inadequate analgesia.

Plastibell size selection is important in male circumcision, which is done by visual estimation of glans size after retraction of the foreskin and clearance of the smegma. Proper fitting is confirmed by applying two to three sizes from the range available. The one that fits appropriately is selected. Numerous complications can occur due to ill-fitting bell size, including proximal migration of the plastic ring and inadequate excision of the foreskin [15].

The thread type and size used have been variable throughout the world, and little research has been done regarding the choice of sutures. One study shows that thinner threads lead to favourable results in terms of the time taken for the bell to fall off [16]. The timing of the bell fall-off has been reported in various studies, although no systematic review has been available to date. One study reported days with a range of 3 to 15 days [16]. Altokhais et al. have concluded that the ring is easy to fall off with polypropylene size 0 suture [17]. In our series, the time to ring fall-off was 7.8±3.04 days with the use of braided nylon thread.

Various complications have been reported with the use of the Plastibell technique. Those include bleeding, infection, proximal migration of the ring, and phimosis. Bleeding usually occurs when the dorsal slit is too lengthy and extends proximally to the application of the ring. Revision of the circumcision with the removal of the ring has been the solution often used [4]. Out of the rest, phimosis needs special attention and happens due to the use of a Plastibell with a lower diameter and tearing of the inner layer of skin with the insertion of the Plastibell. Later on, with cicatrix formation, there is a chance of contraction and phimosis formation. To prevent this, the following points could help: use of an appropriate size Plastibell, taking care not to damage the inner layer of skin, and regular retraction of the outer layer after the ring has fallen off [18]. In our series, none of the subjects developed phimosis.

## Conclusions

Male Circumcision is one of the oldest surgical procedures performed. Several methods are in practice but male circumcision using the Plastibell method is a safe method and is associated with fewer complications. Randomized controlled trials are required to compare the technique to other circumcision methods.
